# Bulk RNA sequencing for analysis of post COVID-19 condition in adolescents and young adults

**DOI:** 10.1186/s12967-024-05117-7

**Published:** 2024-03-26

**Authors:** Silke Lauren Sommen, Zhi Zhao, Sunniva Segtnan, Tonje Stiansen-Sonerud, Joel Selvakumar, Lise Beier Havdal, Johannes Gjerstad, Vegard Bruun Bratholm Wyller, Lise Lund Berven

**Affiliations:** 1 Department of Pediatrics, Akershus University Hospital, Lørenskog, Norway; 2 University of Oslo, Oslo, Norway; 3 Oslo Centre for Biostatistics and Epidemiology (OCBE), Institute of Basic Medical Sciences, Faculty of Medicine, University of Oslo, Oslo, Norway; 4 Department of Clinical Molecular Biology (EpiGen), University of Oslo and Akershus University Hospital, Lørenskog, Norway; 5 Institute of Clinical Medicine, Faculty of Medicine, University of Oslo, Oslo, Norway; 6 Department of Behavioural Sciences, Faculty of Health Sciences, Oslo Metropolitan University, Oslo, Norway

**Keywords:** Post COVID-19 condition, Long COVID, Child, Adolescent, RNA sequencing, Transcriptomics, Immunology

## Abstract

**Background:**

Post COVID-19 condition (PCC) is a complication of SARS-COV-2 infection and can lead to long-term disability.

**Methods:**

The present study was designed to analyse the gene expression patterns of PCC through bulk RNA sequencing of whole blood and to explore the potential molecular mechanisms of PCC. Whole blood was collected from 80 participants enrolled in a prospective cohort study following SARS-CoV-2 infected and non-infected individuals for 6 months after recruitment and was used for bulk RNA sequencing. Identification of differentially expressed genes (DEG), pathway enrichment and immune cell deconvolution was performed to explore potential biological pathways involved in PCC.

**Results:**

We have found 13 differentially expressed genes associated with PCC. Enriched pathways were related to interferon-signalling and anti-viral immune processes.

**Conclusion:**

The PCC transcriptome is characterized by a modest overexpression of interferon-stimulated genes, pointing to a subtle ongoing inflammatory response.

**Supplementary Information:**

The online version contains supplementary material available at 10.1186/s12967-024-05117-7.

## Background

After widespread vaccination campaigns and the emergence of new viral variants, there is a decline in severe COVID-19 cases and related deaths. The focus has therefore shifted from acute COVID-19, a viral disease caused by SARS-CoV-2, to Post COVID-19 condition (PCC). Research efforts are now concentrated on patients that, despite the resolution of the symptoms associated with the acute infection, experience ongoing long-term complications. The prevalence of fatigue after acute COVID-19 ranges from 13 to 33% at 16–20 weeks post disease onset, and is similar to reported fatigue after other acute infections (e.g. Epstein Barr virus infection), which can lead to a diagnosis of post-infective fatigue syndrome (PIFS) [1]. The pathogenesis of PCC and PIFS remains largely unclear, which presents a bottleneck for the development of targeted therapies.

Past research has tended to report on PCC in hospitalized, adult patients. Less attention has been focused on adolescents and young adults with long-lasting symptoms after an initially mild COVID-19 infection, as evidenced by fewer published studies and common limitations in the studies (e.g. absence of control group) [2, 3]. The estimated prevalence of PCC among children and young adults varies from 16.5 to 25.2% [4–6], but greatly depends on both post-infection window and inclusion of a control group. Studies with a COVID-19 negative control group or a longer follow-up time tend to report lower PCC prevalence [7–9]. While studies in adults have identified changes in immune subsets and their transcriptional profiles as common features in PCC [10–12], similar evidence is lacking in young people. Hypothetical PCC mechanisms include persistent antigen reservoirs [13, 14], reactivation of latent viruses [15], increased systemic inflammation and autoimmunity [16].

As ongoing inflammation beyond the acute phase of infection may have implications in potential treatment modalities, a thorough assessment of how the immune system is involved in PCC pathophysiology is required. Whole blood bulk RNA sequencing, a powerful technique for the study of gene expression profiles, is performed here to reveal transcriptional features of PCC in adolescents and young adults. The aims of the study are to identify differences at the transcriptome level which are associated with persistent symptoms after COVID-19 convalescence, and which are not present in individuals experiencing uneventful COVID-19 recovery or in unexposed controls. This study investigates whole blood transcriptomes in a prospective cohort of adolescents and young adults with and without microbiologically confirmed COVID-19 infection and blinded, rigorous PCC case assessment.

## Methods

### Enrolment of participants and collection of samples

Blood samples were obtained from 80 participants enrolled in the Long-Term Effects of COVID-19 in Adolescents and Young Adults (LoTECA) study, a prospective, observational cohort study of non-hospitalized adolescents and young adults testing positive and negative for SARS-CoV-2 (ClinicalTrials.gov identifier: NCT04686734). A detailed recruitment procedure and selected data have been reported elsewhere [17–20]. All participants underwent a one-day investigational program at our study center (Akershus University Hospital, Norway) at inclusion and at 6-month follow-up. The assessment included a standardized medical assessment, symptom surveys, biological sampling, and function testing. In this paper, results from a composite questionnaire containing validated inventories including the Chalder Fatigue Questionnaire[21], the De Paul Symptom Questionnaire [22], the Pediatric Quality of life Inventory (PedsQL) [23], the Hospital Anxiety and Depression Scale [24] and a modified COVID-19 symptom inventory [25] were used to chart fatigue, post-exertional malaise, quality of life, depression/anxiety symptoms and infectious/respiratory/cognitive symptoms respectively [19, 20].

**Fig. 1:**
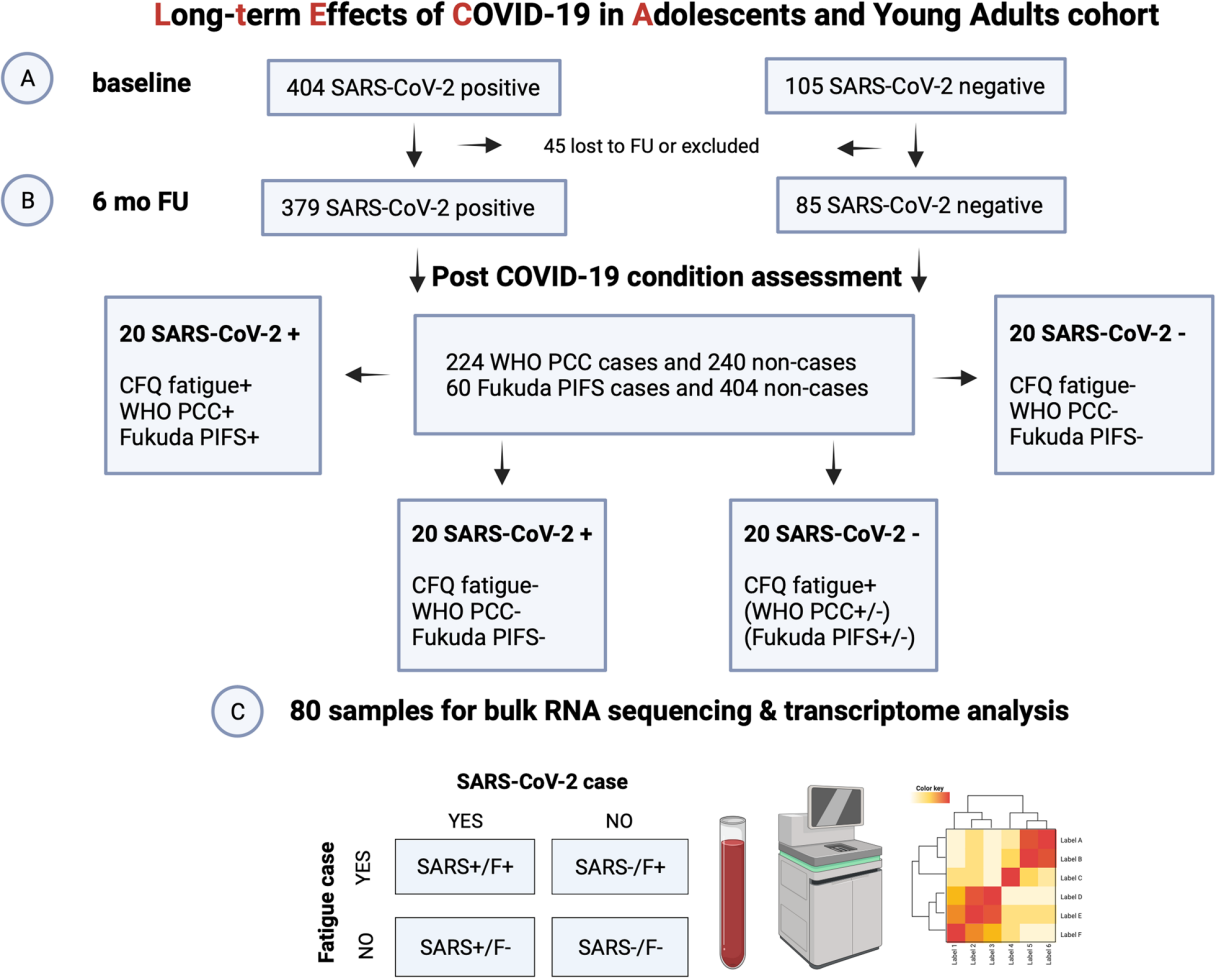
Flow diagram of study design. Participant allocation throughout the study is depicted in Fig. 1: baseline (**A**), follow-up (**B**), transcriptome sequencing analysis (**C**). *WHO* World Health Organization; *PCC* Post COVID-19 Condition; PIFS, post-infectious fatigue syndrome

After the 6-month follow-up, all participants were classified as PCC cases/non-cases and PIFS cases/non-cases according to their adherence to two standardized, operationalized definitions of PCC: the World Health Organization’s definition of Post COVID-19 Condition (PCC) and the Fukuda criteria for post-infectious fatigue syndrome (PIFS) [26, 27]. Classification was carried out rigorously, blinded for initial SARS-CoV-2 status and based on evaluation of all collected data and assessment of medical or psychiatric comorbidity. Of note, SARS-CoV-2 negative participants were subject to testing of nasopharyngeal secretion and serology for SARS-CoV-2; both were confirmed to be negative. The sample selection for this sub study was based on reported fatigue, the hallmark symptom of PCC and PIFS, which was recorded using the Chalder Fatigue Questionnaire (CFQ) [21]. Selection based on CFQ numerical scores allowed for better contrast between the experimental groups with regards to symptom intensity and frequency, with higher scores pointing to higher symptom burden. Twenty SARS-CoV-2 positive participants with high fatigue score (numerical CFQ $\ge 21)$ and adherence to both the WHO PCC and the Fukuda PIFS criteria were selected for bulk RNA sequencing. Additionally, twenty SARS-CoV-2 positive participants with uncomplicated recovery, defined by low fatigue scores (numerical CFQ $\le 11)$ and non-adherence to PCC/PIFS case criteria were included. Furthermore, forty SARS-CoV-2 negative participants were included as controls. The uninfected controls included twenty participants that did not report fatigue and did not adhere to PCC/PIFS case definitions, as well as twenty participants that were identified as fatigue-cases by the Chalder Fatigue Questionnaire (bimodal CFQ $\ge 4)$[21]. Thus, the experiment included four groups: SARS-COV-2 positive cases with fatigue (SARS + F +), SARS-COV-2 positive cases without fatigue (SARS + F-), and SARS-COV-2 negative cases with fatigue (SARS-F +) and without fatigue (SARS-F-) (Fig. 1). All selected participants were female to avoid confounding due to gender differences in differentially expressed genes [28], to take into account a higher PCC prevalence among women [29], and for practical reasons since our cohort was composed of 60% females [19]. This study was approved by the Norwegian National Committee for Ethics in Medical Research and written informed consent was obtained from all participants (or their guardians).

### Transcriptome amplification, library construction and sequencing

Total RNA was isolated from whole blood collected and stored in PAXgene® Blood RNA tubes (BD Bioscience, NJ, USA). RNA purification was performed according to the manufacturer’s protocol using the QIAsymphony SP (QIAGEN, Germany) and the QIAsymphony PAXgene Blood RNA kit (QIAGEN, Germany). RNA quality and concentration were appraised using an Agilent 2100 Bioanalyzer (Agilent Technologies, CA, USA) and NanoDrop (Thermo Fisher Scientific, MA, USA). Preparation of RNA library, transcriptome sequencing using Illumina NovaSeq 6000 (Illumina, CA, USA), read quality assessment, mapping to the human reference genome version GRCh38 (Genome Reference Consortium) and gene expression quantification was conducted by Novogene CO. LTD (Beijing, China). An expression matrix containing counts of 58 735 mRNA-Seq transcripts was used for downstream analyses.

### Statistical analysis

The count data of the mRNA-Seq transcripts were normalized using the Bioconductor package DESeq2 (version 1.42.0) [30]. To identify differentially expressed genes related to PCC based on our prospective cohort design, we used DESeq2 by introducing an interaction term between the SARS-CoV-2 indicator variable (SARS ±) and the fatigue caseness indicator variable (F ±), which adjusts the additional effect of SARS-CoV-2 compared to non-fatigued groups.

$$design = \sim SARS + F + SARS:F$$

To control the false discovery rate, the strictest Bonferroni correction for multiple testing of our 58 735 transcript features is too conservative and has low statistical power, which might be not able to identify truly significant transcript features. To increase the statistical power, we applied the Benjamini–Hochberg correction for multiple testing [31]. Mann Whitney-U test was used for a group comparison of specific DEGs. To understand the biological functionality of the identified differential expressed genes associated with PCC, we performed over-representation analysis (ORA) based on the Kyoto Encyclopedia of Genes and Genomes (KEGG), Gene Ontology (GO) and the Reactome pathway databases using the WEB-based GEne SeT AnaLysis Toolkit (*WebGestalt*) [32]. The WebGestalt 2019 version was used, which includes functional categories KEGG Release 88.2, Gene Ontology accessed on 01/14/2019 and Reactome Version 66. In addition, enrichment analysis of transcription factor binding sites (TFBSs) was performed using the UniBind enrichment tool [33], which can locate interactions between our identified genes and transcription factors to understand transcriptional regulation. For all the enrichment analyses, the FDR was kept under 5% using Benjamini–Hochberg correction for multiple testing.

To study whether differences in relative abundance of distinct cell types are related to PCC, we used CIBERSORTx [34] to estimate cell type proportions. The leukocyte matrix containing 22 cell types blood signature (LM22) for 10 subtypes was used as reference data for gene expression deconvolution. The Dirichlet regression model was applied to jointly analyze the composition of the 10 cell types associated with SARS ± , F + /F- and SARS-fatigue interaction by adjusting age, body-mass index (BMI) and vaccine conditions of participants [35]. BMI, age and infectious agents may affect immune cell composition and may contribute to inter-sample variability [36], confounding the association between PCC and immune cell subsets.

Furthermore, we explored relationships between differentially expressed genes (DEGs) and seven PCC related symptom variables (i.e., fatigue score, post-exertional malaise, cognitive symptoms, respiratory symptoms, symptoms of depression, symptoms of anxiety, quality of life). Group-penalized multiresponse regression with group- and ridge-penalties was used to account for the correlations between the seven symptom variables [37]. *P*-values of the DEGs corresponding to the seven symptom variables were computed using the bootstrap.

## Results

### Participant characteristics

At the time of sampling, the recruited participants were at a mean of 216 days from SARS-COV-2 PCR testing. Mean age was 19.3 years. All participants were female. Participants in the SARS + /F + and SARS-/F + group reported markedly higher fatigue and post exertional malaise than the participants in the SARS + /F- and SARS-/F- groups. Similarly cognitive symptoms, respiratory symptoms and symptoms of anxiety or depression were more common in the SARS + /F + and SARS-/F + group than in the other two groups. The SARS + /F + group reported the lowest quality of life among the four groups. Further details are shown in Table 1.

**Table 1:** Participant characteristics by experimental group

	Group			
	SARS + /F + (n = 20)	SARS + /F- (n = 20)	SARS-/F + (n = 20)	SARS-/F- (n = 20)
Time since symptom onset/PCR test (days, median (IQR))	212 (12)	213 (12)	211 (15)	215 (13)
Demographic characteristics				
Sex (female, %))	20 (100)	20 (100)	20 (100)	20 (100)
Age (median (IQR))	21 (7)	19 (7)	19 (3)	19 (5)
BMI (median (IQR))	21 (6)	21 (4)	23 (6)	23 (4)
Ethnicity (European (%))	14 (70)	15 (75)	20 (100)	20 (100)
Current comorbidity (any (%))	2 (10)	0 (0)	3 (15)	0 (0)
COVID-19 immunization (yes (%))	15 (75)	14 (70)	18 (90)	19 (95)
Symptoms and functional impairment scores^a^				
Fatigue^b^ (score 0–33, mean (SD))	25 (3)	10 (2)	17 (3)	11 (2)
Post exertional malaise^c^ (score 0–100, median (IQR))	73 (26)	0 (5)	23 (23)	0 (6)
Cognitive symptoms^d^ (range 3–15, median (IQR)	13 (3)	3.5 (2)	8.5 (5)	4.5 (2)
Respiratory symptoms^e^ (range 2–10, median (IQR))	5 (2.3)	3 (1)	3 (1)	3 (1)
Symptoms of anxiety^f^ (range 0–21, median (IQR))	10 (6.5)	4 (3.5)	9 (5.3)	4 (3.3)
Symptoms of depression^g^ (range 0–21, median (IQR))	7 (4.3)	1.5 (2.5)	3.5 (6)	1 (2.3)
Quality of life^h^ (range 0–100, median (IQR))	49 (12)	89 (7.6)	70 (15)	87 (11)
Laboratory findings				
Hemoglobin g/dL (mean, SD)	12.73 (0.56)	12.7 (1.50)	13.52 (0.87)	13.42 (0.55)
Leukocyte count, 109 cells/L (mean, SD)	6.5 (1.7)	6.4 (2.2)	6 (1.6)	5.8 (1.5)
hsCRP (plasma, mg/L, median (IQR))	0.8 (0.9)	1.1 (1.1)	2.4 (5.0)	3.5 (4.9)
IP10 (plasma, pg/mL, median (IQR))	124 (56)	112 (78)	98 (70)	91 (77)
Eotaxin (plamsa, pg/mL, median (IQR))	14.2 (4.1)	13.9 (3.8)	11.3 (3.9)	10.1 (6.0)
MCP-1 (plasma, pg/mL, median. (IQR))	4.6 (3.2)	5.2 (3.1)	3.0 (2.7)	1.9 (4.9)
SARS-CoV-2 antibody titer^i^	22 (23)	17 (56)	0.1 (0)	0.1 (0)

*BMI* body mass index; *MCP* monocyte chemotactic protein; *IP* interferon gamma induced protein; *hsCRP* high-sensitive assay of C-reactive protein; *PCR* polymerase chain reaction

^a^with the exception of quality of life, higher values imply more symptoms. For quality of life, higher values imply higher quality of life and less functional impairment

^b^From the Chalder Fatigue Questionnaire

^c^From the DePaul Symptom Questionnaire

^d^The sum score across the 3 items memory problems, concentration problems, and decision-making problems

^e^The sum of scores across dyspnea and coughing

^f^From the Hospital Anxiety and Depression Scale anxiety subscale

^g^From the Hospital Anxiety and Depression Scale depression subscale

^h^From the Pediatric Quality of Life Inventory

^i^Total antinucleocapsid immunoglobulin G and M

### A PCC transcriptional profile characterized by overexpression of 13 genes.

**Fig. 2:**
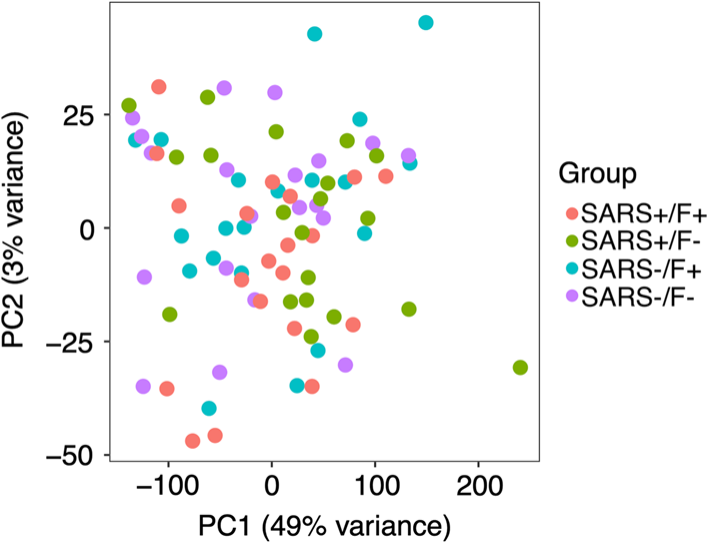
Dimensionality reduction by principal components analysis based on log2 normalized counts of all genes for all participants. Each dot corresponds to the sample of one participant. The smaller the distance between the dots, the greater the similarity of the gene expression profiles. PC1, principal component 1; PC2, principal component 2; percentage expresses contribution to the overall variability in the data

**Fig. 3:**
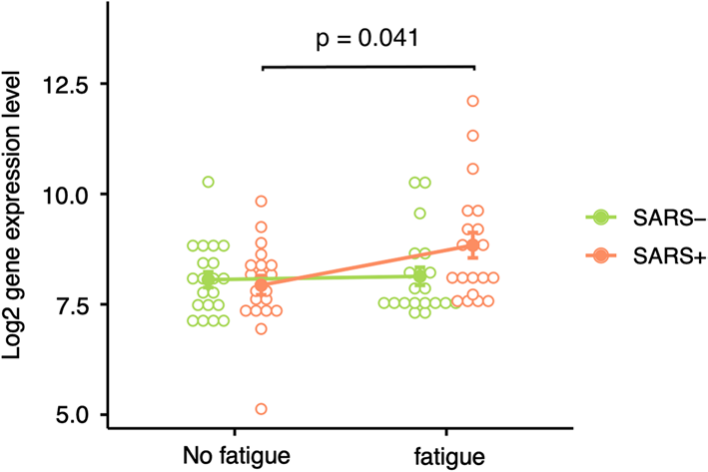
Line plot of expression levels of OAS3, an interferon-inducible antiviral effector protein. The colour indicates SARS-COV-2 infection history: pink = prior SARS-CoV-2 infection, green = no SARS-CoV-2 infection. The groups are further divided based on fatigue symptom load: fatigue versus no fatigue. The *p*-value shows the significance when comparing the expression levels of OAS3 between SARS + subjects with fatigue and no fatigue by Mann Whitney-U test

**Fig. 4:**
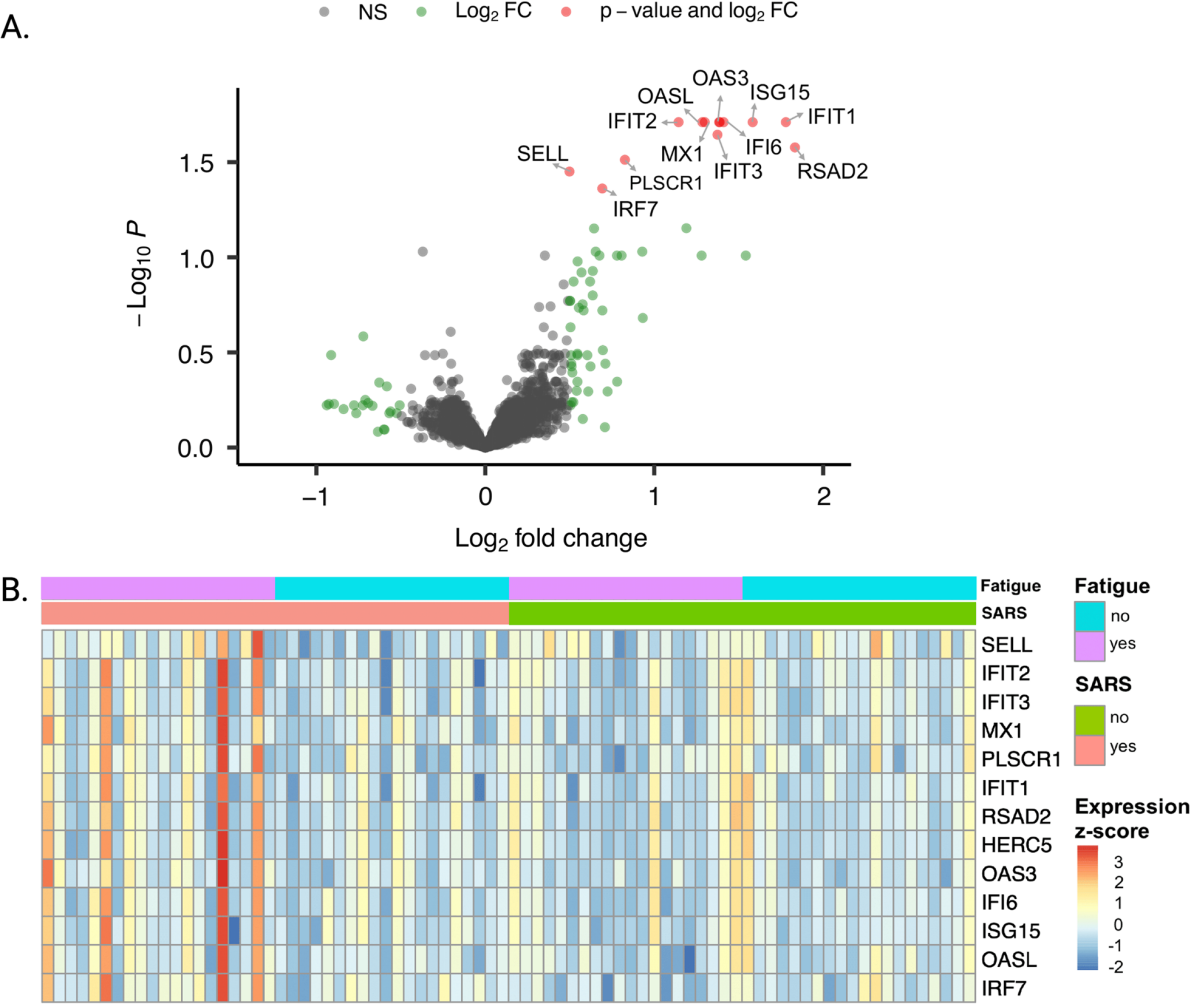
Differential gene expression analysis for participants divided into four experimental groups depending on prior SARS-CoV-2 infection and fatigue symptoms. **A** Volcano plot of differentially expressed genes for participants with prior SARS-CoV-2 infection and fatigue symptoms (SARS + /F +) relatively to fully recovered participants with SARS-CoV-2 infection (SARS + /F-). Genes with significantly increased expression are represented with red dots. The threshold for the adjusted p-value was set to 0.05. The grey dots indicate absolute value of log2 (fold-change) smaller than 0.50. FC, fold change; NS, non-significant. **B** Heat map of differentially expressed genes in participants with prior SARS-CoV-2 infection and fatigue symptoms (SARS + /F +) relative to participants in the other three groups, with raw expression values being scaled

Whole blood derived transcriptome sequencing resulted in 58 735 profiled features. A global overview of the data through principal components analysis (PCA) did not reveal distinct clustering among the four experimental groups (Fig. 2). However, 13 genes were returned as differentially expressed in the SARS + /F + group versus SARS + /F- group after adjustment using the gene expression data from the SARS-/F + and SARS-/F- groups as control data. These DEGs were retained for further analysis. Interestingly, these 13 genes only showed differential expression for participants that reported both fatigue symptoms and a prior SARS-COV-2 infection. The comparisons of SARS + /F- versus SARS-/F- groups and SARS-/F + versus SARS-/F- groups did not show any differentially expressed genes after multiple testing correction. To illustrate, the expression levels of *OAS3*, a member of the OAS antiviral effector protein family, show similar levels between symptomatic and symptom-free participants in the SARS-CoV-2 negative group, but show different levels in the COVID-19 positive group (Fig. 3). The volcano plot, the heat map and the output data table contain the log2 fold-change as well as the adjusted p-values for the 13 DEGs (Fig. 4, Additional file 1: Table S1). Of note, *RSAD2,* an interferon-inducible member of the radical S-adenosylmethionine (SAM) superfamily of enzymes which inhibits viral replication and assembly, was the most upregulated gene and *OASL,* an enhancer of antiviral activity through retinoic acid-inducible gene I (RIG-1)-based signalling, had the lowest p-value [38, 39]. Elevated expression was further found for *IRF7*, a member of the interferon regulatory transcription factor family, and several interferon-stimulated genes (ISGs), including the IFIT family of viral replication inhibitors (*IFIT1, IFIT2, IFIT3*) [40], the ubiquitin ligase *HERC5* responsible for ISGylation of viral target proteins [41], and ubiquitin-like *ISG15* [42]*.* Moreover, we found an upregulation of *IFI6,* an ISG and antiapoptotic protein, which was recently shown to enhance SARS-CoV-2 infection [43]. Other DEGs with immune-related functions include *SELL*, a cell surface adhesion molecule that belongs to a family of adhesion/homing receptors and is required for binding and rolling of leucocytes on endothelial cells [44], *MX1*, a guanosine triphosphate (GTP)-metabolizing anti-viral enzyme [45] and *PLSCR1,* a phospholipid scramblase family member preventing viral entry [46]. None of the identified DEGs in this study were downregulated.

### Enrichment of anti-viral responses in PCC at 6 months after acute SARS-CoV-2 infection

**Fig. 5:**
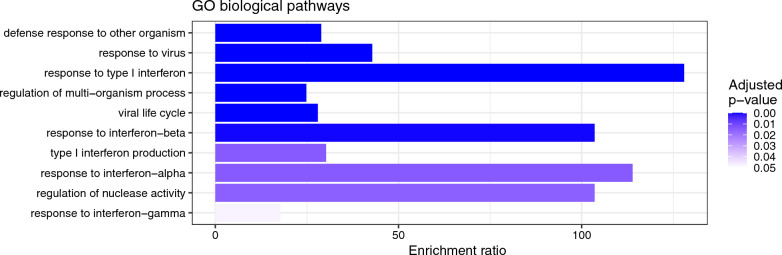
Bar chart of enrichment ratios of significantly enriched Gene ontology (GO) biological pathways. The pathways are ranked by increasing adjusted p-value. The enrichment ratio shows the number of observed divided by the number of expected genes in the gene list of the GO category

**Fig. 6:**
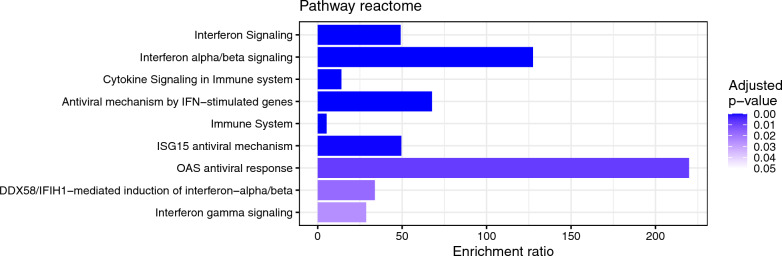
Bar chart of enrichment ratios of significantly enriched KEGG pathways. The pathways are ranked by increasing adjusted p-value. The enrichment ratio shows the number of observed divided by the number of expected genes in the gene list of the KEGG category

**Fig. 7:**
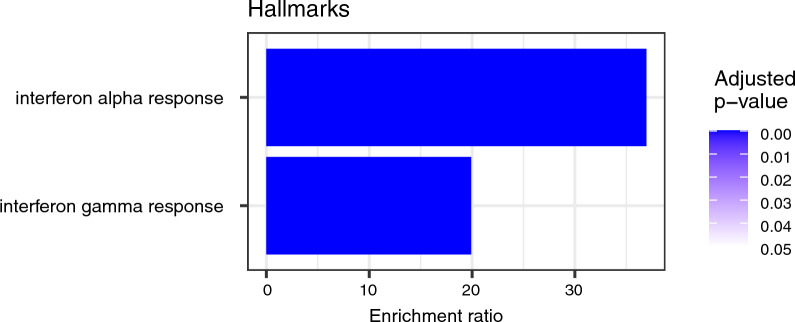
Bar chart of enrichment ratios of significantly enriched Reactome pathways. The pathways are ranked by increasing adjusted p-value. The enrichment ratio shows the number of observed divided by the number of expected genes in the gene list of the Reactome category

To integrate the information obtained from the RNA sequencing output, we performed an enrichment analysis of the DEG list by ORA based on the gene ontology (GO) biological process database. This showed enriched generic responses to viral infections, including several biological categories of interferon-mediated responses (Fig. 5). To further understand the biological meaning of the DEGs, we ran an enrichment analysis using the Kyoto Encyclopedia of Genes and Genomes (KEGG) and Reactome databases. The KEGG enrichment analysis showed, in line with the results of GO enrichment analysis, that the induced genes are primarily found in human viral disease pathways (Fig. 6). Influenza A, hepatitis C and measles viruses infect a host by inhibiting interferon-stimulated gene induction [47]. The Reactome output confirms the common biological theme of interferon related signalling (Fig. 7). The ranked lists of relevant pathways are described in Additional Table 2.

### STAT1/STAT2 are key regulators in the PCC transcriptional profile.

**Fig. 8:**
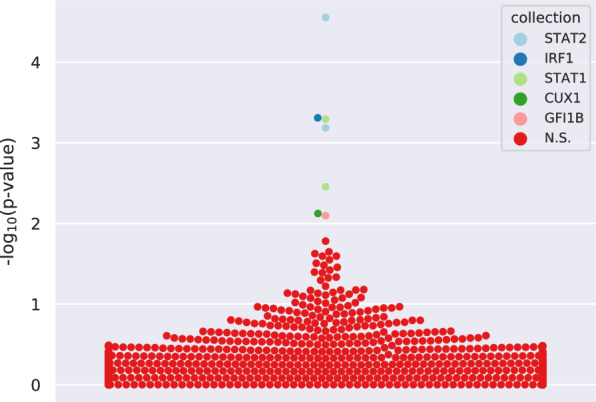
Bee swarm plot to visualize the top 5 enriched transcription factors after transcription factor enrichment analysis using UniBind. Each dot represents a transcription factor; N.S. non-significant

Changes in transcript abundance in bulk sequencing data can be attributed to changes in transcriptional regulation or changes in composition of leucocyte populations. Therefore, a deconvolution of whole blood leucocyte populations was performed “*in silicio*” using CIBERSORTx. This deconvolution into immune cell subsets revealed statistically significant differences in population abundance of monocytes, CD4 memory resting and CD8 cell types between the whole SARS + group and the whole SARS- group, but no evidence of statistical differences related to the SARS + /F + group (Additional file 1: Fig. S1). Transcription factor binding profile enrichment analysis using UniBind, however, revealed five upstream transcription regulators (TF) for the DEG list: *STAT1, STAT2, IRF1, GFI1b, CUX1* (Fig. 8). *STAT1* and *STAT2* belong to the signal transducer and activator of transcription family and play a key role in the immune response against viruses. They are major mediators of the signal from interferons and initiate the transcription of interferon-stimulated genes in the nucleus [48, 49]. A similar function is exerted by *Interferon Regulatory Factor 1 (IRF1)*, a transcription factor induced by interferon I and mediating the pro-inflammatory response of interferon I [50].

### Association of DEG with PCC associated clinical markers.

**Fig. 9:**
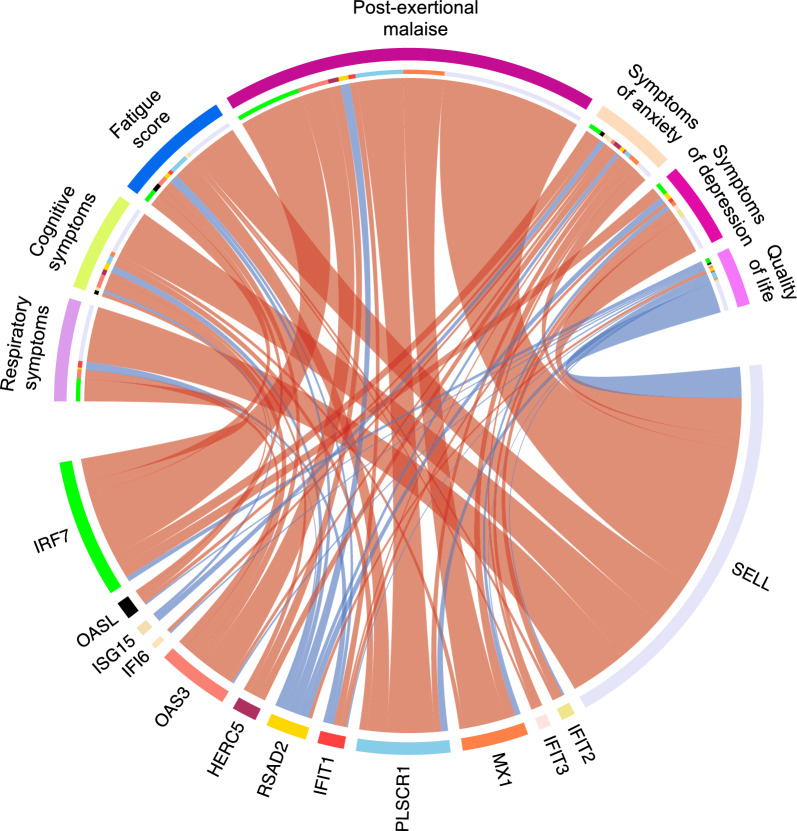
Chord diagram of associations between seven clinical symptoms and 13 DEG expression levels for all samples. Clinical symptoms are represented on the top part of the circle, while DEGs are represented on the bottom part of the circle. The arcs are colour-coded with red for positive effect and blue for negative effect. The width of the arc represents the gene effect on its corresponding clinical symptom. Fatigue score, from the Chalder Fatigue Questionnaire; Post-exertional malaise, from the DePaul Symptom Questionnaire; cognitive symptoms, the sum score across the 3 items memory problems, concentration problems, and decision-making problems; respiratory symptoms, the sum of scores across dyspnea and coughing; symptoms of anxiety, from the Hospital Anxiety and Depression Scale anxiety subscale; symptoms of depression, from the Hospital Anxiety and Depression Scale depression subscale; quality of life, from the Pediatric Quality of Life Inventory

To explore relationships between gene expression and clinical markers, a multivariate multiresponse regression was performed on the 13 DEG expression levels and PCC associated symptoms (fatigue score, post-exertional malaise, cognitive symptoms, respiratory symptoms, symptoms of depression, symptoms of anxiety, quality of life). Several positive correlations between DEG expression levels and clinical symptoms were revealed (Fig. 9, Additional file 1: Table S3). All DEGs, except *RSAD2,* were positively correlated with post-exertional malaise, a hallmark symptom of PCC [51]. As expected, an opposite pattern was shown for quality of life, where higher DEG expression levels were associated with lower quality of life scores. *SELL*, encoding a leukocyte adhesion molecule which regulates leukocyte trafficking to sites of inflammation [52] and which has been linked to severe COVID-19 illness [53], has the largest effect on clinical markers in the correlation analysis. Taken together, these results point to an association between DEG expression and symptom load.

## Discussion

In this study, we identified a PCC-related RNA expression pattern comprising 13 differentially expressed genes. To our knowledge, this is the first comparative analysis of whole blood transcriptomes in adolescents and young adults with PCC after a COVID-19 infection and several control groups including recovered infected, symptomatic uninfected and symptom- free uninfected participants. This design considers that fatigue symptoms in the follow-up period can be a consequence of pandemic living conditions with disruption of routine life, social isolation, and physical distancing rather than a viral infection itself. It takes into account that fatigue symptoms are frequently reported in the general population [54].

The main result from the study was a PCC-related transcriptional upregulation in interferon signalling related genes. These genes were not upregulated in uninfected controls with fatigue symptoms or in recovered SARS-CoV-2 infected participants. SARS-CoV-2 is an RNA virus, which upon entry into the host cell, triggers a cellular host defence mechanism mediated by pattern recognition receptors (PRRs) that induce interferon production. Interferons initiate the transcription of interferon-stimulated genes (ISGs), a group of anti-viral effector proteins. Several members of ISGs were identified as differentially expressed in our RNA sequencing analysis. These DEGs play a critical role in the initial resistance to the SARS-CoV-2 viral infection. However, upregulation of these transcripts at six months after the initial SARS-CoV-2 infection points to aberrant activation of antiviral innate immunity and interferon induction possibly due to a chronic SARS-CoV-2 infection or a sustained inflammatory response after clearance of the virus. In fact, low-grade inflammation in SARS-CoV-2 infected participants was previously reported in our study cohort, although no link with PCC could be established [20]. Evidence for viral persistence or insufficient clearance of viral remnants after SARS-CoV-2 infection has been hypothesized in studies showing ongoing evolution of specific immunity [12, 13, 55], elevated anti-viral immunity [11] and persistent viral antigen presence [14, 56–58]. The observed immune perturbation could also be the downstream consequence of reactivation of latent viruses following acute SARS-CoV-2 infection (e.g. Epstein Barr virus) [15, 59]. Transcripts like *OAS3* and *MX1* specifically act to inhibit viral replication, while other transcripts also have non-immune related roles in other cellular processes and diseases, such as *ISG15* in DNA repair [60]. Thus, despite a functional enrichment analysis restricted to anti-viral responses, non-immune mechanisms might also be at play. It is possible that interferon is at the centre of the disease process, even if the PCC affected do not have a detectable serum IFN level. In systemic lupus erythematosus (SLE), for instance, transcriptome analysis also shows the absence of interferon I/II/III gene transcripts, but interferon still plays an important role in the pathogenesis of the disease as evidenced by an abundance of upregulated interferon-inducible genes [61]. Potentially, the cells producing interferon have migrated to distant sites of injury and are not captured by the blood sample used for gene expression analysis.

Our findings show similarities with earlier human and animal PCC studies. Persistently elevated expression of IFN was reported in different human post COVID-19 cohort studies [11, 55]. Woodruff et al. show evidence for persistent viral triggering of de novo B cell response and IFN-γ signalling in PCC [62], while others have found prolonged CD8 + T cell-mediated IFN-γ release in PCC [63]. Additionally, SARS-CoV-2 infected hamsters had persistent inflammation with upregulation of IFN in the olfactory tissue, which was accompanied by behavioural change [64]. However, Berentschot et al. show a reduction of IFN I/II and a non-activation of ISGs in monocytes in PCC patients, suggesting an impairment of the innate anti-viral response [65]. Similarly, decreased expression of multiple IFN-I-inducible genes including *MX1*, *OAS3* and *OASL* has been associated with persistent symptoms after COVID-19 [66]. Yin et al. report a lack of major perturbations in their transcriptomics study, but present elevated frequencies of T cell subpopulations and increased exhaustion markers in individuals with PCC, indicating an ongoing stimulation with viral antigens at 8 months post-infection [67]. A longitudinal study of COVID-19 sequelae, however, found no evidence of persistent viral infection, autoimmunity, or abnormal immune activation in PCC [68]. These large discrepancies between studies could be attributed to differences in cohorts and assays or to complex immune disturbances in PCC. Our findings can serve as a starting point to further explore immunological aberrations in PCC.

How immune perturbations contribute to PCC-related symptoms remains largely unanswered. Reduced circulating serotonin was recently proposed as an explanation for neurocognitive symptoms in PCC, where serotonin depletion was caused by type I IFN induction by viral RNA [69]. Increased frequencies of IFN-γ-producing SARS-CoV-2-specific T cells were also suggested as drivers of prolonged dyspnoea in PCC [70], and a recent gene expression study shows increased regulation of IFN production and T cell activation in PCC affected with brain fog [71]. Although causal conclusions cannot be made from our cross-sectional design, the present data suggests a positive association between differential gene expression and clinical symptoms, especially post-exertional malaise. This observation warrants further studies as the small to moderate fold changes in the SARS + /F + group are unlikely to explain the severe symptom burden. Earlier findings from our cohort show that PCC symptoms are associated to psychosocial factors (eg. loneliness) rather than the SARS-CoV-2 infection itself [19]. Interestingly, this aligns with the “Conserved Transcriptional Response to Adversity (CTRA)”, a concept describing stress-induced changes in immune related gene expression (72). The biological factors in PCC, such as immune activation, might be the result of a conditioning mechanism by chronic stress in addition to the SARS-COV-2 infection. Our findings share similarities with  observations made in PIFS: Higher IFN gamma production was observed in Q fever fatigue syndrome patients after ex vivo stimulation of whole blood with *Coxiella burnetti*, but there was no correlation between these findings and the level of fatigue or symptom duration [73]. The clinical overlap between PCC and PIFS could mean that gene expression correlates are shared independent of the infectious trigger. Indeed, some studies report evidence for immune dysregulation with increased IFN signaling and viral innate immune enhancement in chronic fatigue patients [74–78], while others do not find overlapping biomarkers between PIFS from different pathogens [79]. We suggest that PCC, like PIFS, is not a primary inflammatory disorder and a simple change in gene expression level is unlikely to explain PCC pathophysiology.

Caution is warranted since our study has several limitations. The results require validation in independent cohorts and currently are of limited applicability to the general population. We focused on female, non-hospitalized adolescents and young adults infected with the B 1.1.7 variant, while the majority of PCC patients were infected with other variants of concern, are older and were hospitalized for severe acute COVID-19. Additionally, our study is based on a small sample size of 80 participants and the participants differed slightly on potential confounding variables. For instance, the SARS-/F + group were less severely fatigued than the SARS + /F + group (median fatigue score 17 versus 25). If immune alterations were due to fatigue causing inflammatory responses rather than the infection itself, this could be an important confounder. Furthermore, we have not found changes in cell population abundance related to the SARS + /F + group, possibly due to the poor quality of CIBERSORTx deconvolution based on our bulk mRNA sequencing data and Dirichlet regression that does not characterize the impact of outliers. Ideally, one would use single cell RNA sequencing to capture gene expression in individual immune cells and more advanced statistical methods for differential discovery, e.g. scCODA and sccomp [80] Nevertheless, however exploratory, our study contributes to the understanding of PCC pathophysiology and can help the future development of studies, prevention, and treatment strategies.

## Conclusion

To conclude, our results suggest that subtle differences in the expression levels of innate immunity related genes, including higher expression of genes involved in interferon signalling, may point to sustained, low-grade inflammation in PCC affected adolescents and young adults.

### Supplementary Information

**Additional file 1: Table S1.** Differentially expressed genes for SARS+/F+ group versus SARS+/F- group. **Table S2.** Pathway enrichment analysis for differentially expressed genes in SARS+/F+ versus SARS+/F-. **Table S3.** Effects (mean effect (standard deviation; *p*-value)) of the DEGs on the symptom variables based on group-penalized multiresponse regression. The standard deviations and *p*-values were computed using 1000 bootstrap replications. **Figure S1.** CIBERSORTx cell-type deconvolution violin plots showing the percentage of cells per group for each of the 10 cell types extracted from the bulk RNA seq data. Dirichlet regression was used to identify differential composition of cell types.

## Data Availability

The datasets and code scripts supporting the conclusions of this article will be available from the corresponding author on request.

## Abbreviations

PCC: Post COVID-19 condition
PIFS: Post-infective fatigue syndrome
DEG: Differentially expressed gene
CFQ: Chalder Fatigue Questionnaire
ORA: Over-representation analysis
KEGG: Kyoto Encyclopedia of Genes and Genomes
GO: Gene Ontology
TFBS: Transcription factor binding site
BMI: Body-mass index
PCA: Principal components analysis
PRR: Pattern recognition receptor
IFN: Interferon
SLE: Systemic lupus erythematosus
CTRA: Conserved transcriptional response to adversity
