# Supplementary material for: Bulk RNA sequencing for analysis of post COVID-19 condition in adolescents and young adults
Source: J Transl Med. 2024 Mar 26;22:312. doi: 10.1186/s12967-024-05117-7 (PMC10964710; doi:10.1186/s12967-024-05117-7)
Supplement: Supplementary file 1 — Additional file 1: Table S1. Differentially expressed genes for SARS+/F+ group versus SARS+/F- group. Table S2. Pathway enrichment analysis for differentially expressed genes in SARS+/F+ versus SARS+/F-. Table S3. Effects (mean effect (standard deviation; p-value)) of the DEGs on the symptom variables based on group-penalized multiresponse regression. The standard deviations and p-values were computed using 1000 bootstrap replications. Figure S1. CIBERSORTx cell-type deconvolution violin plots showing the percentage of cells per group for each of the 10 cell types extracted from the bulk RNA seq data. Dirichlet regression was used to identify differential composition of cell types. [file 12967_2024_5117_MOESM1_ESM.docx]

**Additional materials**

Table S1. Differentially expressed genes for SARS+/F+ group versus SARS+/F- group.

| **Ensembl. ID** | **Gene name** | **p-value** | **adj. p-value** | **Log2 fold-change** |
| --- | --- | --- | --- | --- |
| ENSG00000119922 | IFIT2 | 1.99e-05 | 0.019 | 1.14 |
| ENSG00000157601 | MX1 | 2.14e-05 | 0.019 | 1.30 |
| ENSG00000185745 | [IFIT1](https://www.proteinatlas.org/ENSG00000185745-IFIT1) | 1.54e-05 | 0.019 | 1.78 |
| ENSG00000138646 | [HERC5](https://www.proteinatlas.org/ENSG00000138646-HERC5) | 1.71e-05 | 0.019 | 1.41 |
| ENSG00000111331 | [OAS3](https://www.proteinatlas.org/ENSG00000111331-OAS3) | 9.39e-06 | 0.019 | 1.38 |
| ENSG00000126709 | [IFI6](https://www.proteinatlas.org/ENSG00000126709-IFI6) | 1.77e-05 | 0.019 | 1.39 |
| ENSG00000187608 | [ISG15](https://www.proteinatlas.org/ENSG00000187608-ISG15) | 8.70e-06 | 0.019 | 1.58 |
| ENSG00000135114 | [OASL](https://www.proteinatlas.org/ENSG00000135114-OASL) | 7.36e-06 | 0.019 | 1.28 |
| ENSG00000119917 | IFIT3 | 2.80e-05 | 0.023 | 1.37 |
| ENSG00000134321 | [RSAD2](https://www.proteinatlas.org/ENSG00000134321-RSAD2) | 3.63e-05 | 0.026 | 1.83 |
| ENSG00000188313 | PLSCR1 | 4.64e-05 | 0.031 | 0.83 |
| ENSG00000188404 | SELL | 5.83e-05 | 0.035 | 0.50 |
| ENSG00000185507 | IRF7 | 7.76e-05 | 0.044 | 0.69 |

Table S2. Pathway enrichment analysis for differentially expressed genes in SARS+/F+ versus SARS+/F-

| **KEGG Categories** | | | | | | | |
| --- | --- | --- | --- | --- | --- | --- | --- |
| **Gene Set** | **Description** | **Size** | **Expect** | **Ratio** | **P Value** | **FDR** | **geneID** |
| hsa05164 | Influenza A | 171 | 0.18814 | 21.260 | 0.000019210 | 0.0062623 | OAS3/RSAD2/MX1/IRF7 |
| hsa05160 | Hepatitis C | 131 | 0.14413 | 20.814 | 0.00029961 | 0.033298 | OAS3/IRF7/IFIT1 |
| hsa05162 | Measles | 132 | 0.14523 | 20.656 | 0.00030642 | 0.033298 | OAS3/MX1/IRF7 |
| **GO biological components** | | | | | | | |
| **Gene Set** | **Description** | **Size** | **Expect** | **Ratio** | **P Value** | **FDR** | **geneID** |
| GO:0098542 | defense response to other organism | 473 | 0.41528 | 28.896 | <2.2e-16 | <2.2e-16 | OAS3/IFIT3/IFIT2/IFI6/RSAD2/OASL/HERC5/MX1/IRF7/IFIT1/ISG15/PLSCR1 |
| GO:0009615 | response to virus | 319 | 0.28007 | 42.846 | <2.2e-16 | <2.2e-16 | OAS3/IFIT3/IFIT2/IFI6/RSAD2/OASL/HERC5/MX1/IRF7/IFIT1/ISG15/PLSCR1 |
| GO:0034340 | response to type I interferon | 89 | 0.078139 | 127.98 | <2.2e-16 | <2.2e-16 | OAS3/IFIT3/IFIT2/IFI6/RSAD2/OASL/MX1/IRF7/IFIT1/ISG15 |
| GO:0043900 | regulation of multi-organism process | 367 | 0.32221 | 24.828 | 1.5252e-10 | 3.2410e-8 | OAS3/RSAD2/OASL/HERC5/MX1/IFIT1/ISG15/PLSCR1 |
| GO:0019058 | viral life cycle | 285 | 0.25022 | 27.975 | 1.4137e-9 | 2.4034e-7 | OAS3/RSAD2/OASL/MX1/IFIT1/ISG15/PLSCR1 |
| GO:0035456 | response to interferon-beta | 33 | 0.028973 | 103.55 | 0.0000028410 | 0.00040248 | IFIT3/IFIT1/PLSCR1 |
| GO:0032606 | type I interferon production | 113 | 0.099210 | 30.239 | 0.00011707 | 0.014215 | HERC5/IRF7/ISG15 |
| GO:0035455 | response to interferon-alpha | 20 | 0.017559 | 113.9 | 0.00013400 | 0.014237 | IFIT3/IFIT2 |
| GO:0032069 | regulation of nuclease activity | 22 | 0.019315 | 103.55 | 0.00016275 | 0.015371 | OAS3/OASL |
| GO:0034341 | response to interferon-gamma | 192 | 0.16857 | 17.797 | 0.00055779 | 0.047412 | OAS3/OASL/IRF7 |
| **Reactome pathways** | | | | | | | |
| **Gene Set** | **Description** | **Size** | **Expect** | **Ratio** | **P Value** | **FDR** | **geneID** |
| R-HSA-913531 | Interferon signaling | 197 | 0.22401 | 49.104 | <2.2e-16 | <2.2e-16 | OAS3/IFIT3/IFIT2/IFI6/RSAD2/OASL/HERC5/MX1/IRF7/IFIT1/ISG15 |
| R-HSA-909733 | Interferon alpha/beta signaling | 69 | 0.078461 | 127.45 | <2.2e-16 | <2.2e-16 | OAS3/IFIT3/IFIT2/IFI6/RSAD2/OASL/MX1/IRF7/IFIT1/ISG15 |
| R-HSA-1280215 | Cytokine signaling in immune system | 688 | 0.78234 | 14.060 | 9.4746e-13 | 5.4574e-10 | OAS3/IFIT3/IFIT2/IFI6/RSAD2/OASL/HERC5/MX1/IRF7/IFIT1/ISG15 |
| R-HSA-1169410 | Antiviral mechanism by IFN-stimulated genes | 78 | 0.088695 | 67.647 | 1.1961e-10 | 5.1670e-8 | OAS3/OASL/HERC5/MX1/IFIT1/ISG15 |
| R-HSA-168256 | Immune system | 1997 | 2.2708 | 5.2844 | 2.0529e-9 | 7.0947e-7 | OAS3/IFIT3/IFIT2/IFI6/RSAD2/OASL/HERC5/MX1/IRF7/IFIT1/ISG15/SELL |
| R-HSA-1169408 | ISG15 antiviral mechanism | 71 | 0.080735 | 49.545 | 8.9407e-7 | 0.00025749 | HERC5/MX1/IFIT1/ISG15 |
| R-HSA-8983711 | OAS antiviral response | 8 | 0.0090969 | 219.85 | 0.000033065 | 0.0081624 | OAS3/OASL |
| R-HSA-168928 | DDX58/IFIH1-mediated induction of interferon-alpha/beta | 78 | 0.088695 | 33.824 | 0.000081460 | 0.017595 | HERC5/IRF7/ISG15 |
| R-HSA-877300 | Interferon gamma signaling | 92 | 0.10461 | 28.677 | 0.00013327 | 0.025587 | OAS3/OASL/IRF7 |

Table S3. Effects (mean effect (standard deviation; *p*-value)) of the DEGs on the symptom variables based on group-penalized multiresponse regression. The standard deviations and *p*-values were computed using 1000 bootstrap replications.

|  | **Respiratory**  **symptoms** | **Cognitive**  **symptoms** | **Fatigue score** | **Post exertional**  **malaise** | **Symptoms of anxiety** | **Symptoms of depression** | **Quality of life** |
| --- | --- | --- | --- | --- | --- | --- | --- |
| **intercept** | -1.446 (0.3957; <0.001) | -0.600 (0.3836; 0.114) | -0.203 (0.2957; 0.479) | -7.283 (1.0079; <0.001) | 0.341 (0.3678; 0.333) | -0.424 (0.663; 0.512) | 5.776 (0.2107; <0.001) |
| **Age** | -0.012 (0.0013; <0.001) | -0.006 (0.0018; 0.003) | -0.001 (0.0011; 0.631) | -0.031 (0.0052; <0.001) | -0.014 (0.0021; <0.001) | -0.029 (0.0024; <0.001) | 0.003 (0.0008; <0.001) |
| **BMI** | 0.004 (0.0008; <0.001) | 0.002 (0.0015; 0.224) | 0.002 (0.001; 0.110) | 0.002 (0.0045; 0.668) | 0.011 (0.0015; <0.001) | -0.001 (0.0022; 0.597) | 0.001 (0. 0008; 0.122) |
| **COVID-19 vaccine** | 0.012 (0.0122; 0.315) | -0.043 (0.0207; 0.039) | -0.010 (0.0127; 0.434) | -0.169 (0.0539; 0.004) | 0.019 (0.0221; 0.401) | -0.012 (0.0262; 0.649) | 0.013 (0.0098; 0.163) |
| **COVID-19 infection** | 0.078 (0.0082; <0.001) | 0.055 (0.0133; 0.001) | 0.080 (0.0103; <0.001) | 0.271 (0.0379; <0.001) | 0.032 (0.0143; 0.028) | 0.196 (0.0181; <0.001) | -0.048 (0.0066; <0.001) |
| **SELL** | 0.158 (0.0105; <0.001) | 0.136 (0.0144; <0.001) | 0.134 (0.0097; <0.001) | 0.398 (0.0407; <0.001) | 0.038 (0.0163; 0.025) | 0.102 (0.0245; <0.001) | -0.086 (0.0081; <0.001) |
| **IFIT2** | 0.007 (0.0044; 0.107) | 0.002 (0.0047; 0.729) | 0.009 (0.0034; 0.020) | 0.015 (0.0126; 0.201) | 0.000 (0.0044; 0.991) | 0.022 (0.0071; 0.003) | -0.005 (0.0025; 0.043) |
| **IFIT3** | -0.001 (0.0031; 0.678) | -0.006 (0.0032; 0.062) | 0.007 (0.0027; 0.010) | -0.002 (0.0089; 0.781) | 0.009 (0.0031; 0.009) | 0.016 (0.0053; 0.002) | 0.000 (0.0017; 0.877) |
| **MX1** | 0.005 (0.0033; 0.130) | 0.013 (0.0039; 0.002) | -0.002 (0.0028; 0.369) | 0.114 (0.0101; <0.001) | 0.030 (0.0043; <0.001) | -0.012 (0.0061; 0.042) | -0.003 (0.0017; 0.046) |
| **PLSCR1** | 0.008 (0.0065; 0.200) | 0.021 (0.0069; 0.005) | 0.054 (0.0051; <0.001) | 0.132 (0.0191; <0.001) | 0.018 (0.0071; 0.002) | 0.019 (0.0114; 0.080) | -0.019 (0.0037; <0.001) |
| **IFIT1** | -0.018 (0.0026; <0.001) | 0.004 (0.0028; 0.173) | -0.011 (0.0021; <0.001) | 0.020 (0.0078; 0.012) | 0.006 (0.0025; 0.036) | 0.011 (0.0046; 0.026) | -0.004 (0.0014; 0.016) |
| **RSAD2** | -0.005 (0.0026; 0.038) | -0.019 (0.0029; <0.001) | -0.015 (0.002; <0.001) | -0.028 (0.0075; 0.002) | -0.012 (0.0027; 0.001) | -0.018 (0.0046; 0.001) | 0.009 (0.0014; <0.001) |
| **HERC5** | -0.002 (0.0034; 0.674) | 0.015 (0.0032; 0.001) | 0.003 (0.0023; 0.153) | 0.029 (0.0088; 0.003) | 0.021 (0.0032; <0.001) | 0.010 (0.0057; 0.071) | -0.002 (0.0017; 0.149) |
| **OAS3** | 0.025 (0.0038; <0.001) | 0.038 (0.0041; <0.001) | 0.026 (0.0029; <0.001) | 0.085 (0.0109; <0.001) | 0.010 (0.0045; 0.031) | 0.001 (0.0071; 0.904) | -0.005 (0.0019; 0.014) |
| **IFI6** | 0.002 (0.0033; 0.465) | 0.003 (0.0036; 0.327) | -0.005 (0.0031; 0.127) | 0.003 (0.0096; 0.752) | 0.010 (0.003; 0.004) | 0.010 (0.0056; 0.074) | -0.005 (0.0018; 0.014) |
| **ISG15** | 0.004 (0.0035; 0.220) | -0.009 (0.0034; 0.013) | -0.002 (0.0025; 0.423) | -0.013 (0.0091; 0.155) | -0.017 (0.0033; 0.001) | -0.004 (0.0055; 0.426) | -0.002 (0.0016; 0.283) |
| **OASL** | -0.001 (0.0035; 0.723) | 0.010 (0.004; 0.019) | 0.021 (0.0034; <0.001) | 0.015 (0.0116; 0.167) | 0.011 (0.0043; 0.012) | -0.009 (0.0063; 0.141) | -0.005 (0.0021; 0.031) |
| **IRF7** | 0.064 (0.0073; <0.001) | 0.015 (0.009; 0.095) | 0.036 (0.0058; <0.001) | 0.182 (0.0246; <0.001) | 0.035 (0.007; <0.001) | 0.034 (0.0134; 0.021) | -0.013 (0.0041; 0.004) |


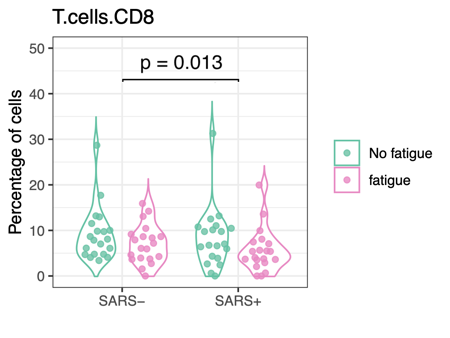


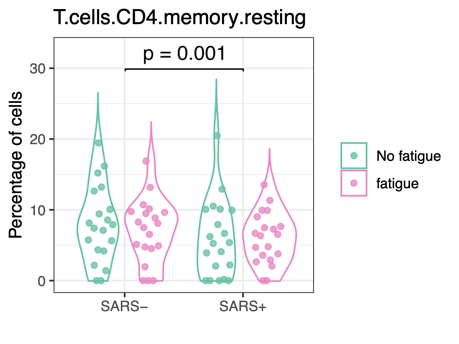


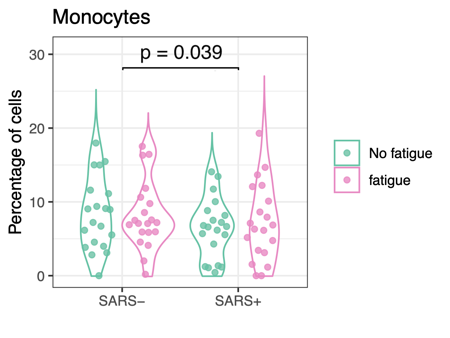

# Figure S1. CIBERSORTx cell-type deconvolution violin plots showing the percentage of cells per group for each of the 10 cell types extracted from the bulk RNA seq data. Dirichlet regression was used to identify differential composition of cell types.
